# Widespread Intra- and Inter-Network Dysconnectivity among Large-Scale Resting State Networks in Schizophrenia

**DOI:** 10.3390/jcm12093176

**Published:** 2023-04-28

**Authors:** Bei Rong, Huan Huang, Guoqing Gao, Limin Sun, Yuan Zhou, Ling Xiao, Huiling Wang, Gaohua Wang

**Affiliations:** 1 Department of Psychiatry, Renmin Hospital of Wuhan University, Wuhan 430060, China; 2 Institute of Neuropsychiatry, Renmin Hospital of Wuhan University, Wuhan 430060, China; 3 Institute of Psychology, CAS Key Laboratory of Behavioral Science, Beijing 100101, China; 4 Taikang Center for Life and Medical Sciences, Wuhan University, Wuhan 430071, China

**Keywords:** schizophrenia, resting-state networks, intra-network functional connectivity, inter-network functional connectivity

## Abstract

Schizophrenia is characterized by the distributed dysconnectivity of resting-state multiple brain networks. However, the abnormalities of intra- and inter-network functional connectivity (FC) in schizophrenia and its relationship to symptoms remain unknown. The aim of the present study is to compare the intra- and inter-connectivity of the intrinsic networks between a large sample of patients with schizophrenia and healthy controls. Using the Region of interest (ROI) to ROI FC analyses, the intra- and inter-network FC of the eight resting state networks [default mode network (DMN); salience network (SN); frontoparietal network (FPN); dorsal attention network (DAN); language network (LN); visual network (VN); sensorimotor network (SMN); and cerebellar network (CN)] were investigated in 196 schizophrenia and 169-healthy controls. Compared to the healthy control group, the schizophrenia group exhibited increased intra-network FC in the DMN and decreased intra-network FC in the CN. Additionally, the schizophrenia group showed the decreased inter-network FC mainly involved the SN-DMN, SN-LN and SN-CN while increased inter-network FC in the SN-SMN and SN-DAN (*p* < 0.05, FDR-corrected). Our study suggests widespread intra- and inter-network dysconnectivity among large-scale RSNs in schizophrenia, mainly involving the DMN, SN and SMN, which may further contribute to the dysconnectivity hypothesis of schizophrenia.

## 1. Introduction

Schizophrenia (SCZ) is a devastating neuropsychiatric disorder affecting 1% of the general population worldwide [1]. Positive symptoms, negative symptoms and impairments in motivation and cognition characterize SCZ [2]. However, to date, the pathophysiological mechanisms of SCZ are largely unclear.

Resting-state functional magnetic resonance imaging (rs-fMRI) presents a compelling framework for understanding the pathophysiology of SCZ. In the explanation of the neurophysiological mechanisms of SCZ, the disconnection hypothesis is widely accepted [3,4,5]. This hypothesis suggests that SCZ is characterized by aberrations between resting-state networks (RSNs) [6,7,8]. RSNs, a term that refers to multiple, spatially independent brain seeds capable of coherent signal fluctuations without specific tasks or stimuli, are involved in processing internal stimuli, executing various higher-level cognitive functions and detecting and integrating salient external stimuli and internal mental processes [8,9,10,11]. Functional connectivity (FC), which is quantified by calculating correlations between fMRI time courses throughout the brain, can be utilized to describe the distribution of intrinsic RSNs in psychiatric disorders [11,12,13]. Deciphering how disturbances of disrupted brain areas operating within large-scale brain networks are essential for understanding the pathophysiology underlying psychiatric disorders.

Brain regions can be arranged in functional networks, and this could revolutionize the way we classify psychiatric disorders from symptom-based to network-based [14]. A growing body of FC evidence suggests that the disconnection of SCZ involves alters in the coupling within spatially distributed large-scale RSNs [15,16,17,18]. The disruption of intra-network FC within multiple RSNs has been demonstrated in SCZ, including the default mode network (DMN) [11], salience network (SN) [19] and frontoparietal network (FPN) [20] and cerebellar network (CN) [21]. However, functional integration can be characterized only partially based on intra-network connectivity, considering high-order cognitive and affective processes typically rely on the dynamic interactions of multiple networks [22]. Several studies also characterized inter-network integration by computing the correlation between average time series across different RSNs. For example, Menon et al. [7] first proposed the triple-network model, focusing on disrupting coupling among DMN, central executive network (CEN) and SN to assess the dynamics of the networks in psychosis [23,24]. Previous findings have shown that the aberrant anterior insula aspect of the SN is critical for the interaction of the DMN and CEN in SCZ, and the decreased activity of this seed is associated with hallucinations [25]. Chang et al. [26] demonstrated that the subsystem of DMN showed disrupted higher FC with the FPN in SCZ patients compared to HC. A longitudinal study focusing on the triple-network connectivity patterns in SCZ found that SN-centered hypoconnectivity was associated with long-lasting negative symptoms after 6 weeks of follow-up [24]. In addition, some studies also investigated the association between these three networks and other RSNs, including the dorsal attention network (DAN) [27,28] sensorimotor network (SMN) [29], visual network (VN) [30], CN [31,32] and auditory network [33]. However, the results of the study concerning the large-scale connectivity abnormalities were mixed, and there was no agreement on the conclusions. For example, some studies revealed increased FC within the DMN [26]. In contrast, other researchers reported both decreased [34,35] and no significantly-altered FC [19,36] within the DMN. These inconsistent results could be attributed to relatively insufficient sample sizes and methodological variations, limiting the confidence in their findings. Thus, assessing the replicability of scientific findings is essential for establishing the robustness of knowledge. In addition, the majority of prior studies have focused on the higher-order RSNs [15,36,37], the VN, SMN and CN, which are known to support more specialized, externally-driven functions, are also equally important in schizophrenia [38,39]. Thus far, few studies have evaluated the functional integration between the networks presented above. In the present study, a relatively larger rs-fMRI dataset was used to investigate the abnormal FC pattern within and between the eight RSNs (i.e., DMN, FPN, SN, DAN, SMN, AN, VN, and CN) in SCZ. Furthermore, the association between aberrant FC of RSNs and clinical variables was explored in SCZ to identify the characteristic alterations of RSNs and shed light on the pathophysiology of SCZ for identifying biomarkers for SCZ.

## 2. Materials and Methods

### 2.1. Participants

A total of 196 patients with SCZ were recruited from the Department of Psychiatry, Renmin Hospital of Wuhan University. Experienced clinicians diagnosed patients with SCZ based on the structured clinical interview according to the Diagnostic and Statistical Manual of Mental Disorders, Fourth Edition (DSM-IV). The inclusion criteria for SCZ were as follows: (1) 18–45 years of age; (2) Han Chinese; (3) right-handed; (4) at least 9 years of education; (5) total score of Positive and Negative Syndrome Scale (PANSS) ≥60; The exclusion criteria for the patients were as follows: (1) a history of electroconvulsive therapy (ECT); (2) diagnoses of other mental disorders; (3) a history of a neurological disorder or severe head injury; (4) the presence of other serious physical illness.

A total of 169 healthy controls (HCs) were recruited for this study. The inclusion and exclusion criteria were similar to those for patients, except that they would be excluded if they or their first-degree relatives met any diagnosis of a psychiatric disorder according to the DSM-IV criteria. This research program was approved by the ethics committee of the Renmin Hospital at Wuhan University.

### 2.2. Clinical Assessments

All patients were assessed during the acute phase of the illness. The severity of the clinical symptoms in the patients with SCZ was estimated using the PANSS Scale. All patients received atypical antipsychotic medicine at the time of scanning, and an equivalent dosage of chlorpromazine was calculated for each day’s antipsychotic medication (mg/day).

### 2.3. Imaging Data Acquisition and Preprocessing

All fMRI scans were performed on GE 3.0 T Signa HDxt scanner (General Electric, Milwaukee, WI, USA) with an 8-channel radio frequency head coil in the Department of Radiology at the Renmin Hospital of Wuhan University. While undergoing the rs-fMRI scan (8′10″ duration), participants were instructed to keep their eyes closed, stay relaxed, and not think about anything. The resting-state fMRI images were obtained using a gradient echo-planar imaging (EPI) sequence (repetition time (TR) = 2 s, echo time (TE) = 30 ms, Field of view (FOV) = 220 mm × 220 mm, flip angle (FA) = 90°, matrix = 64 × 64, slice thickness = 4 mm, slice gap = 0.6 mm, 240 volumes). T1-weighted high-resolution data (Structural images) were acquired using an MPRAGE sequence (TR = 7.8 ms, TE = 3.0 ms, FOV = 220 mm × 220 mm, FA = 7°, matrix = 256 × 256, slice thickness = 1 mm, slice gap = 1 mm, 188 volume).

We analyzed all image data using MATLAB (Mathworks, Inc., Natick, MA, USA) and the CONN toolbox v.19c [40]. For longitudinal magnetization stability, the first 5 time points of functional data were discarded. Preprocessing included the functional realignment and unwrapping, slice-timing correction, direct functional segmentation and normalization to Montreal Neurological Institute (MNI) space and structure normalization to MNI space, and functional spatial smoothing with a 6-mm full width at half maximum (FWHM) kernel. All images were converted into standard stereotaxic space and resampled at 2 × 2 × 2 mm^3^ voxel size. The outliers were identified using the ART toolbox if they were more than 3 standard deviations away from the mean image intensity. The anatomical component base noise reduction strategy (aCompCor) estimates spurious sources of noise (like physiological effects) [41]. In the denoising step, BOLD signals were deconfounded using aCompCor, scrubbing (identified outliers by ART), and motion regression. Then, a default band-pass filter was applied with a frequency window of 0.008–0.09 Hz.

### 2.4. ROI-to-ROI Whole Brain Functional Connectivity Analysis

**Figure 1:**
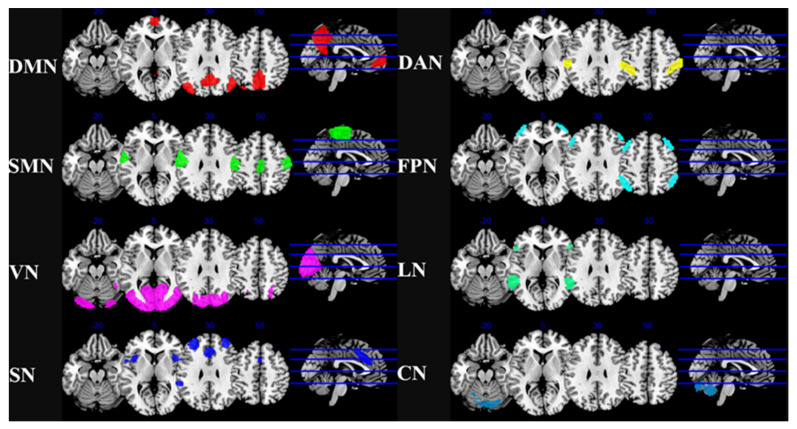
Resting-state brain network map. Abbreviation: DMN, default mode network; SMN, sensorimotor network; VN, visual network; SN, salience network; DAN, dorsal attention network; FPN, frontoparietal network; LN, language network; CN, cerebellar network.

The RSNs were defined using validated independent component analysis (ICA) templates from the HCP (Human Connectome Project), which had supported by empirical data [40,42,43]. We selected the 8 main RSNs were used as the networks of interest, including 32 ROIs representing DMN, FPN, SN, DAN, LN, VN, SMN, and CN. In each ROI, the rs-fMRI time series were calculated by averaging all voxels within each seed. The correlation coefficients of Pearson were calculated between the seed time series and those of all other voxels. To increase normality, Fisher’s r-to-z transform was applied. Spatial maps of the 8 RSNs and the coordinates of 32 ROIs are described in Table 1 and Figure 1.

### 2.5. Statistical Analysis

Demographic and clinical variables were evaluated using independent samples *t*-tests (2-tail) or Chi-square (χ^2^) tests. A 2-sample *t*-test was used to determine whether ROI-to-ROI FC varied between the groups. In the analysis, gender, age, education level, and mean FD at baseline were fitted as covariates. For false-positive results, multiple comparisons were performed with the connection level false-discovery rate (FDR)-corrected *p* < 0.05.

The mean FC z-values in the patients were obtained from the clusters that showed a significant difference in FC results between the 2 groups. A partial correlation analysis was conducted to further test the association between the FC differences and various clinical variables (the Scale for the Assessment of Negative Symptoms (SANS), the Scale for the Assessment of Positive Symptoms (SAPS), the Scale for the Assessment of general psychopathology symptoms, PANSS total scores, duration of illness and chlorpromazine equivalents (CPZ)) after controlling for gender, age, education years and mean FC in the SCZ group. Benjamini–Hochberg false discovery rate (FDR) < 0.05 was used for multiple comparisons.

## 3. Results

### 3.1. Demographics and Clinical Characteristics

The demographic and clinical characteristics of the two groups are listed in Table 2. The demographic and clinical characteristics of the two groups are listed in Table 2. Two-sample *t*-tests and chi-square (χ^2^) tests, respectively, revealed no significant group differences with respect to age and gender composition (all *p* > 0.05). The SCZ group had lower educational levels (*p* < 0.001) and larger head motion (*p* = 0.048) than the HC group.

### 3.2. Overall Characteristics of Intra- and Inter-Network Connectivity

**Figure 2:**
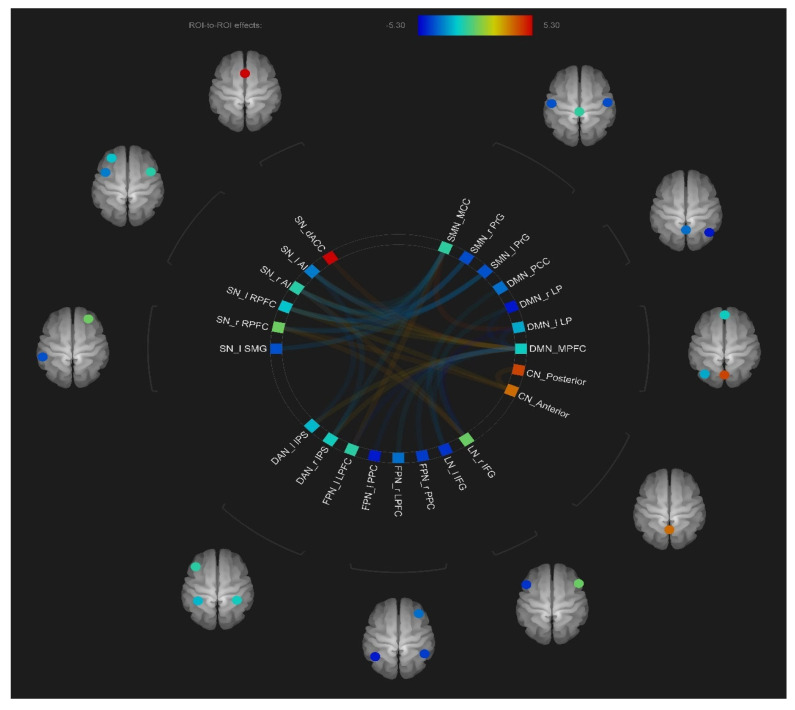
The intra− and inter−connectivity of the eight resting−state brain networks. Abbreviation: DMN, default mode network; FPN, frontoparietal network; SN, salience network; DAN, dorsal attention network; LN, language network; SMN, sensorimotor network; CN, cerebellar network. l, left; r, right; MPFC, medial prefrontal cortex; LP, lateral parietal; LPFC, lateral prefrontal cortex; PPC, posterior parietal cortex; AI, anterior insula; RPFC, rostral prefrontal cortex; IPS, intraparietal sulcus; PCC, posterior cingulate cortex; IFG, inferior frontal gyrus; FEF, frontal eyes field; dACC, dorsal anterior cingulate cortex; SMG, supramarginal gyrus, MCC, middle cingulate cortex; PrG, precentral gyrus. Each node represents each subregion of resting−state networks. The lines connecting two ROIs represent the degree of connectivity strength differences between schizophrenia patients and healthy controls. Warm color represents lower connectivity in schizophrenia patients than healthy controls, and cool color indicates higher connectivity in SCZ patients than HCs. Darker color indicates a larger strength difference.

As shown in Figure 2, a connectogram of the RSN subregions was utilized to illustrate the group differences in intra- and inter-network connectivity.

#### 3.2.1. Intra-Network Connectivity

As shown in Figure 2 and Table 3, compared with the HC group, the SCZ group showed significantly increased FC between MPFC and bilateral LP within the DMN (t = −3.68, FDR-corrected *p* < 0.01; t = −4.1, FDR-corrected *p* < 0.01, respectively). However, the SCZ group showed significantly reduced FC between the anterior lobules and posterior lobules within the compared CN (t = 3.13, FDR-corrected *p* < 0.05). No significant differences were found within the SN, FPN, SMN, DAN, LN and VN between the two groups (FDR-corrected *p*-values > 0.05).

#### 3.2.2. Inter-Network Connectivity

**Figure 3:**
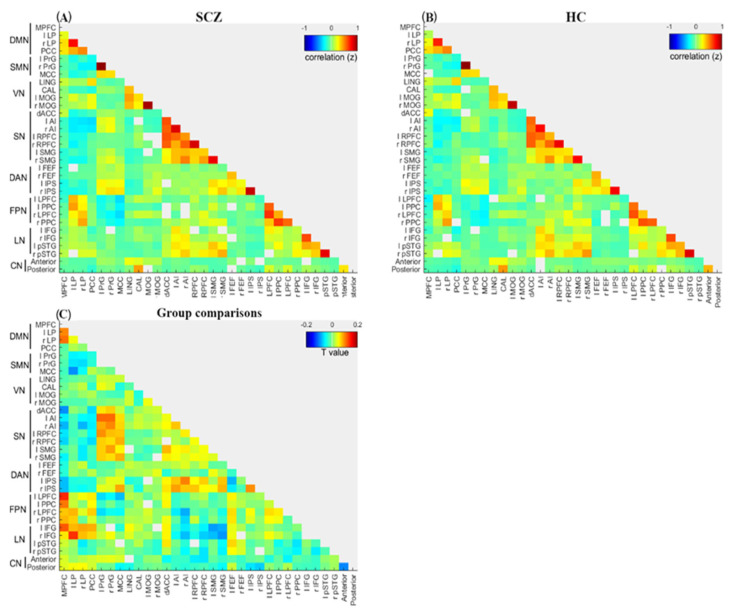
Inter−network connectivity matrices. Inter−network connectivity matrix for the (**A**) Schizophrenia patients and (**B**) Healthy controls. Network nodes in the functional connectivity matrix correspond to the 32 regions of interest (ROIs) from the eight RSNs. The correlation coefficient (Fisher’s z) represents the inter−network connectivity between the 32 ROIs. Warm color denotes positive connectivity, and cold color denotes negative connectivity. (**C**) Group difference of mean functional connectivity between schizophrenia patients and healthy controls (T−value). Warm color represents decreased connectivity in schizophrenia patients compared to the healthy controls, and cool color represents increased connectivity in schizophrenia patients compared to the healthy controls. Abbreviation: l, left; r, right; MPFC, medial prefrontal cortex; LP, lateral parietal; PPC, posterior parietal cortex; PrG, precentral gyrus; MCC, middle cingulated cortex; LING, Lingual gyrus; CAL, calcarine; MOG, middle occipital gyrus; dACC, dorsal Anterior cingulate cortex; AI, anterior insula; RPFC, rostral prefrontal cortex; SMG, supramarginal gyrus; FEF, frontal eye field; IPS, intraparietal sulcus; LPFC, lateral prefrontal cortex; PPC, posterior parietal cortex; IFG, inferior frontal gyrus; pSTG, posterior superior temporal gyrus; Anterior, Cerebellar anterior lobules; Posterior, Cerebellar posterior lobules.

As shown in Figure 3 and Table 3, compared to the HC group, the SCZ group ex-hibited a significantly increased inter-network between MPFC and bilateral LPFC (t = −4.4, FDR-corrected *p* < 0.001; t = −3.77, FDR-corrected *p* < 0.01, respectively), between MPFC and bilateral PCC (t = −3.24, FDR-corrected *p* < 0.001; t = −2.62, FDR-corrected *p* < 0.05, respectively), between MPFC and left IFG (t = −3.65, FDR-corrected *p* < 0.01), be-tween PCC and left IFG (t = −3.01, FDR-corrected *p* < 0.05), between bilateral LP and right IFG (t = −5.3, FDR-corrected *p* < 0.001; and t = −2.95, FDR-corrected *p* < 0.05, respectively), between right AI and left IPS (t = −3.6, FDR-corrected *p* < 0.05), between left AI and bilateral PrG (t = −3.21, FDR-corrected *p* < 0.01; t = −3.73, FDR-corrected *p* < 0.01, respectively), between right AI and bilateral PrG (t = −3.11, FDR-corrected *p* < 0.05; t = −2.8, FDR-corrected *p* < 0.05, respectively), between bilateral AI and MCC (t = −2.82, FDR-corrected *p* < 0.05; t = −2.55, FDR-corrected *p* < 0.05, respectively), between left RPFC and bilateral PrG (t = −3.55, FDR-corrected *p* < 0.05; t = −2.87, FDR-corrected *p* < 0.05, respectively), right RPFC and bilateral PrG (t = −2.77, FDR-corrected *p* < 0.05; t = −2.87, FDR-corrected *p* < 0.05, respectively), between bilateral RPFC and MCC (t = −2.83, FDR-corrected *p* < 0.05; t = −3.31, FDR-corrected *p* < 0.05, respectively), between left SMG and bilateral PrG (t = −2.9, FDR-corrected *p* < 0.05; t = −3.35, FDR-corrected *p* < 0.05, respectively), and between MCC and right IPS (t = −2.76, FDR-corrected *p* < 0.05).

Conversely, compared to the HC group, the SCZ group showed significantly decreased inter-network connectivity between MPFC and bilateral AI (t = 2.56, FDR-corrected *p* < 0.05; t = 2.89, FDR-corrected *p* < 0.05, respectively), between MPFC and dACC (t = 3.87, FDR-corrected *p* < 0.01), between MPFC and left RPFC (t = 2.39, FDR-corrected *p* < 0.05), between MPFC and bilateral IPS (t = 2.39, FDR-corrected *p* < 0.05; t = 2.65, FDR-corrected *p* < 0.05, respectively), between left LP and MCC (t = 4.19, FDR-corrected *p* < 0.001), between bilateral RPFC and anterior lobules (t = 2.77, FDR-corrected *p* < 0.05; t = 3.34, FDR-corrected *p* < 0.05, respectively), and between MCC and left LPFC (t = 3.18, FDR-corrected *p* < 0.05).

### 3.3. Relationships between Functional Connectivity Patterns and Clinical Variables

No significant associations were detected between clinical variables (SAPS, SANS, general psychopathology subscales scores of the PANSS, total scores of the PANSS, duration of illness and CPZ) and the altered FC of intra- or inter-network connectivity after FDR multiple corrections (all FDR-corrected *p*-values > 0.05). The partial correlation between clinical variables and the altered FC of intra- or inter-network connectivity are described in Supplementary Table S1 and Figure S1.

## 4. Discussion

This study comprehensively investigated the intra- and inter-network FC of eight RSNs (i.e., DMN, SN, FPN, DAN, SMN, VN, LN and CN) in a relatively larger SCZ dataset and showed that SCZ patients exhibited broad aberrations within and between-networks. Specifically, increased intra-network FC in the DMN and decreased intra-network in the CN were found in SCZ patients compared to HCs. Furthermore, SCZ patients displayed inter-network functional dysconnectivity in multiple networks, including DMN, SN, SMN and CN. The findings indicated that SCZ has fundamental abnormalities integration within the high, low-order, and between them, supporting the disconnection hypothesis of SCZ.

We characterized functional integration within networks and found aberrant intra-network FC in DMN and CN. The DMN is one of the principal components of the brain’s functional architecture and is involved in endogenously generated thought, autobiographical memory, and self-referential and conceptual processing [44,45]. In the current study, increased FC was detected between MPFC and bilateral LP within DMN; this result was in line with the previous findings [46]. The connectivity increased within the DMN may indicate excessive concentration on interoceptive thought as well as a disturbance in SCZ [22]. The most common finding is that of enhanced intra-network of DMN in resting states [24,47]. Some studies identified the non-affected relatives and those at high psychotic risk also exhibited hyperconnectivity in the DMN [48,49], indicating that this feature may represent an endophenotype of SCZ.

Traditionally, it has been assumed that the cerebellum is solely responsible for motor learning and coordination. This view, however, has been challenged by the increasing recognition of the cerebellum participating in cognitive and affective processes. Exner et al. [50] found that the anterior cerebellar regions are associated with motor function, while the posterior cerebellar lobules are principally tied to cognitive functions. In recent years, mounting neuroimaging evidence reported the structural and functional anomalies of the cerebellum in SCZ [21,51]. Herein, we observed that the patients with SCZ have decreased intra-network connectivity between the anterior and posterior lobules of the CN compared to HCs, as described previously [37,52]. The altered FC within the CN was considered to drive many cognitive deficits in SCZ [53].

Since the brain function is determined by multiple distributed networks and not an individual brain network, the inter-network FC among large-scale RSNs was also investigated in this study. Subsequently, the DMN exhibited increased FC with the FPN and decreased FC with the SN in SCZ compared to HCs. The SN is involved in monitoring, processing and integrating the salient external emotional cues and internal events [54,55]. The reduced inter-network FC between the MPFC of the DMN and the key hubs (dACC and bilateral AI) of the SN is similar to the previous studies that reported decreased FC between DMN and SN in SCZ [56,57]. During self-referential processing, the MPFC has long been recognized as the key region of coding self-relevance and salience attribution [58,59]. As the most prominent hubs of SN, AI is responsible for receiving convergent information input from the visual and auditory cortex [60,61] and has been uniquely associated with introspection, the awareness of the body’s internal emotional response and cognitive states [62]. The abnormal FC between the MPFC of DMN and SN in SCZ might be related to pathophysiological disturbance of salience attribution [63]. The decreased FC between SN and DMN is specific that can distinguish SCZ from other psychotic diseases, such as obsessive-compulsive disorder [57], depression [64] and bipolar disorder [65]. The disruption of communication between the SN and DMN might contribute to positive symptoms of SCZ, as described previously [66]. At the same time, we did not find a significant correlation between the symptoms and aberrant inter-network FC between SN and DMN. This might be due to such correlations can be blurred by the duration of illness and antipsychotic medication [67].

FPN is involved in decision-making processes and is pivotal in goal-directed cognition by flexibly coupling with either the default or dorsal attention network [68]. Accumulating evidence suggested the FPN and DMN interaction to monitor and adjust self-related thinking consistent with goals and task demands [34,69]. In the present study, we found increased inter-network connectivity between the MPFC of the DMN and the seeds of the FPN, including bilateral PCC and LPFC. These results are partially consistent with the previous findings [70,71]. The changes suggested that the abnormal interactions of DMN and FPN may be the foundation of impaired coordination between self-monitoring and task performance, a core signature of SCZ [72]. Interestingly, two studies found increased connectivity between DMN and RFPN [30,73] and decreased connectivity between DMN and LFPN [71]. This partial inconsistency may be due to the diversity of methodology or the smaller sample size. Thus, whether there is an abnormal lateralization of FPN connectivity with DMN in SCZ needs to be explored further.

The aberrant inter-network FC between DMN and FPN caters to an influential triple network model in psychopathology [25,58]. Traditional views proposed that abnormalities integration of DMN-FPN rely on the aberrant input for the AI [74], suggesting that the aberrant emotional salience processing can be used to interpret the disruption integration of self-monitoring and task performance in psychotic disorder [75]. However, in this study, we did not find any altered interconnectivity between the SN and FPN in SCZ. Similarly, we only observed aberrant FC within the DMN but not within the SN or the FPN. Thus, these null findings might be interpreted by the paradigm of the resting state, which mainly engages internally-directed processes charged by the DMN. In agreement with this explanation, the dysfunction of the SN and the FPN is usually reported in the state of external stimuli task. These findings were consistent with recent research [36,75] and those reporting dysfunction of the FPN or SN in SCZ patients under resting state [19,20,76]. Nonetheless, this hypothesis needs to be investigated, and the effects of different experimental paradigms (for example, resting state vs. task) need to be examined on the outcomes.

By integrating sensory, visceral, and affective information, the SN is believed to direct attention and shape cognitive and behavioral responses [55,77]. In the present study, we found that the right AI of SN exhibited increased inter-network with the left IPS of DAN. The SN is mainly involved in stimulus-driven (bottom-up) attention and cognitive control [78], while the DAN mainly includes the FEF and IPS and is engaged by goal-directed (top-down) attention [79]. The SN and DAN are activated during a series of tasks involving externally oriented attention [77,79] and switch attention and reallocate focus, processes that contribute to SCZ [77,80,81]. Functional integration abnormalities between SN and DAN have been reported predominantly in task-fMRI studies of SCZ [69,82,83] and fewer studies in resting-state [84,85]. Lefort et al. [84] found that the right AI of SN showed decreased FC with the right DLPFC of DAN in a multisite dataset. One meta-analysis did not find the aberrant rsFC between the SN and DAN [85]. These results differed from the finding in this study. The inconsistencies between the results are due to the heterogeneity of SCZ patients. Thus, the disrupted inter-network between the SN and DAN may be contributed to the dysfunction of the attentional switching and reallocation in SCZ.

Furthermore, we found that the SN seeds were hyperconnected with the SMN, which is engaged in the perception of external stimuli. Salience detection is maintained by the SN by receiving and integrating input from sensory areas and by interacting with the SMN [86]. The disruption to SN networks impairs the appropriate assessment of salience and leads to distorted perceptions of reality [7]. Consequently, an imbalance in communication between the SN and SMN may Contribute to sensory processing anomalies in SCZ. Recently, Bulbul et al. [87] reported that the SN exhibited an enhanced inter-network with SMN might be an endophenotype candidate for SCZ. Additionally, we demonstrate that significantly decreased inter-connectivity between the bilateral RPFC of SN and the anterior of CN, suggesting the disruption of the cerebellar-subcortical-cortical loop in SCZ. The present results were supported by cognitive dysmetria, implicating that cognitive and affective impairment is linked to the cerebellum and its connections with the prefrontal cortex (during the cerebello-thalamo-cortical circuit) [88,89]. Combined with the alterations within the cerebellum, this finding indicated that cerebellar dysfunction in both internal and external models might be the pathology underlying SCZ.

The SMN is involved in the specialized processing of sensory stimuli and motor responses and consists of postcentral gyrus, precentral gyrus, and supplementary motor area [90]. The DMN is associated with internally-directed processes, including conceptual processing, self-monitoring, and autobiographical and spontaneous cognition [44,45,91]. FPN (some studies termed it CEN) [55,92,93] consists of DLPFC and PPC and is engaged in executive functions and adaptive cognitive control [94]. SN is speculated to be engaged in response to interceptive awareness, task-set maintenance, and the detection of salient external stimuli [55]. DAN anchors IPS and FEF and is involved in top-down attentional-control processes [95]. The four latter networks carry out a distinctive role, and their interactions subserve cognitive control, and all of these networks were termed “high-order cognitive functional networks” [92,93]. Specifically, SMN exhibited increased inter-network FC with the right AI region of the SN and the left IPS of DAN as well as decreased inter-network FC between the MPFC of the DMN cortex and the left LPFC of the FPN, suggesting extensive disturbance integration between the lower-level sensory system and high-order cognitive functional network in SCZ. These connectivity changes have also been reported in previous studies [22,92]. These extensive disruptions might contribute to the inability to integrate top-down regulation with bottom-up sensory input, resulting in deficits of high-order cognitive functions, which were regarded as the core components of SCZ [96]. Overall, this study added to the evidence suggesting pathological interaction between SMN and high-order cognitive networks of SCZ [18,97,98]. Gao et al. [99] used a support vector machine (SVM) to determine whether the brain activity in the SCZ differentiated from that in the HC and identified that the functional activity of SMN was a classifier with an accuracy of 98.13%. Thus, we inferred that SCZ might be characterized by functional abnormalities of the SMN. Future investigations of these processes are warranted to further unveil the underlying etiology of various aspects of psychopathology.

Nevertheless, the present study has some limitations. First, most of the SCZ patients had a long course of disease before enrollment, and many patients were prescribed antipsychotic treatment before enrollment. Thus, the potential effects of medication and clinical course on brain activity cannot be excluded [100]. In the future, first-episode drug-naïve SCZ patients would be enrolled to eliminate medication and clinical course effects on functional integration of RSNs. Second, the HC group had a higher education level than the SCZ group. Thus, to reduce the influence of education level, the HC group was education level-matched with the SCZ group. Further studies should recruit first-episode drug-naive SCZ patients and education-level-matched HCs.

## 5. Conclusions

In summary, the current study showed the widespread intra- and inter-network dysconnectivity among large-scale resting state networks in SCZ, mainly involving the DMN, SN, SMN, and CN, which may further contribute to the dysconnectivity hypothesis of the disorder.

## Figures and Tables

**Table 1:** Montreal Neurological Institute (MNI) coordinates of thirty-two ROIs in the resting-state brain networks.

		MNI Coordinates		
Name of RSNs	Regions of Interests (ROIs)	x	y	z
Default mode network (DMN)				
	Medial prefrontal cortex (MPFC)	1	55	−3
	Left lateral parietal (lLP)	−39	−77	33
	Right lateral parietal (rLP)	47	−67	29
	Posterior cingulate cortex (PCC)	1	−61	38
Sensorimotor network (SMN)				
	Left precentral gyrus (lPrG)	−55	−12	29
	Right precentral gyrus (rPrG)	56	−10	29
	Middle cingulate cortex (MCC)	0	−31	67
Visual network (VN)				
	Lingual gyrus (LING)	2	−79	12
	Calcarine sulcus (CAL)	0	−93	−4
	Left middle occipital gyrus (lMOG)	−37	−79	10
	Right middle occipital gyrus (rMOG)	38	−72	13
Salience network (SN)				
	dorsal Anterior cingulate cortex (dACC)	0	22	35
	Left anterior insula (lAI)	−44	13	1
	Right anterior insula (rAI)	47	14	0
	Left rostral prefrontal cortex (lRPFC)	−32	45	27
	Right rostralprefrontal cortex (rRPFC)	32	46	27
	Left supramarginal Gyrus (lSMG)	−60	−39	31
	Right supramarginal Gyrus (rSMG)	62	−35	32
Dorsal attention network (DAN)				
	Left frontal eye field (lFEF)	−27	−9	64
	Right frontal eye field (rFEF)	30	−6	64
	Left intraparietal sulcus (lIPS)	−39	−43	52
	Right intraparietal sulcus (rIPS)	39	−42	54
Frontoparietal network (FPN)				
	Left lateral prefrontal cortex(lLPFC)	−43	33	28
	Right lateral prefrontal cortex(rLPFC)	41	38	30
	Left posterior parietal cortex (lPPC)	−46	−58	49
	Right posterior parietal cortex (rPPC)	52	−52	45
Language network (LN)				
	Left inferior frontal gyrus (lIFG)	−51	26	2
	Right inferior frontal gyrus (rIFG)	54	28	1
	Left posterior superior temporal gyrus (lpSTG)	−57	−47	15
	Right posterior superior temporal gyrus (rpSTG)	59	−42	13
Cerebellar network (CN)				
	Cerebellar anterior lobules (Anterior)	0	−63	−30
	Cerebellar posterior lobule (Posterior)	0	−79	−32

**Table 2:** Demographic and clinical characteristics.

	SCZ	HC	*p* Value
	(n = 196)	(n = 169)	
Gender (male/female)	98/98	85/84	0.955 ^a^
Age (years)	25.41 ± 5.63	25.01 ± 4.91	0.63 ^b^
Education (years)	12.42 ± 2.78	14.79 ± 2.17	<0.001
Mean FD (mm)	0.08 ± 0.06	0.07 ± 0.05	0.048
PANSS			
Total	82.70 ± 11.62	—	—
Positive symptoms	21.21 ± 4.67	—	—
Negative symptoms	20.15 ± 5.67	—	—
General psychopathology symptoms	41.20 ± 6.81	—	—
CPZ equivalents (mg/d)	373.75 ± 283.66	—	—
Duration of illness (months)	46.23 ± 54.06	—	—

Note: mean FD, mean frame-wise displacement; PANSS, Positive and Negative Symptom Scale; ^a^, Chi-square test; ^b^, two-sample *t*-test; —, no value. Abbreviation: SCZ, schizophrenia patients; HC, healthy controls.

**Table 3:** Aberrant functional connectivity of intra-networks and inter-networks between two groups.

Significant Connections			Functional Connectivity		T	*p* Value
Network	Seed Seed	Target Seed	SCZ	HC		
			Mean (SD)	Mean (SD)		
Intra-network Connectivity						
DMN	MPFC	l LP	0.2 (0.27)	0.1 (0.25)	−3.68	<0.01
	MPFC	r LP	0.32 (0.25)	0.22 (0.24)	−4.1	<0.01
CN	Anterior	Posterior	0.3 (0.25)	0.4 (0.22)	3.13	<0.05
Inter-network Connectivity						
DMN-FPN	MPFC	l LPFC	−0.14 (0.24)	−0.28 (0.22)	−4.4	<0.001
	MPFC	r LPFC	−0.05 (0.24)	−0.12 (0.23)	−3.77	<0.01
	MPFC	l PPC	−0.06 (0.24)	−0.17 (0.22)	−3.24	<0.01
	MPFC	r PPC	−0.01 (0.24)	−0.07 (0.23)	−2.62	<0.05
DMN-SN	MPFC	dACC	0.13 (0.25)	0.23 (0.24)	3.87	<0.01
	MPFC	l AI	0.15 (0.24)	−0.09 (0.23)	2.56	<0.05
	MPFC	r AI	−0.13 (0.25)	−0.04 (0.23)	2.89	<0.05
	MPFC	L RPFC	−0.08 (0.26)	−0.02 (0.22)	2.39	<0.05
DMN-DAN	MPFC	l IPS	−0.21 (0.22)	−0.14 (0.23)	2.39	<0.05
	MPFC	r IPS	−0.19 (0.22)	−0.11 (0.22)	2.65	<0.05
DMN-LN	MPFC	l IFG	−0.02 (0.26)	−0.14 (0.23)	−3.65	<0.01
	PCC	l IFG	−0.14 (0.20)	−0.23 (0.21)	−3.01	<0.05
	l LP	r IFG	−0.11 (0.22)	−0.03 (0.19)	−5.3	<0.001
	r LP	r IFG	0.07 (0.25)	−0.01 (0.22)	−2.95	<0.05
DMN-SMN	l LP	MCC	−0.21 (0.19)	−0.11 (0.18)	4.19	<0.001
SN-DAN	r AI	l IPS	0.05 (0.24)	−0.05 (0.22)	−3.6	<0.05
SN-SMN	l AI	l PrG	0.19 (0.26)	0.08 (0.21)	−3.21	<0.01
	l AI	r PrG	0.23 (0.25)	0.12 (0.20)	−3.73	<0.01
	l AI	MCC	−0.01 (0.21)	−0.05 (0.19)	−2.82	<0.05
	r AI	l PrG	0.16 (0.24)	0.08 (0.22)	−3.11	<0.05
	r AI	r PrG	0.24 (0.25)	0.14 (0.25)	−2.8	<0.05
	r AI	MCC	0.02 (0.23)	−0.04 (0.20)	−2.55	<0.05
	l RPFC	l PrG	−0.04 (0.21)	−0.13 (0.19)	−3.55	<0.05
	l RPFC	r PrG	−0.01 (0.20)	−0.09 (0.19)	−2.87	<0.05
	l RPFC	MCC	−0.03 (0.21)	−0.1 (0.19)	−2.83	<0.05
	r RPFC	l PrG	−0.02 (0.21)	−0.09 (0.20)	−2.77	<0.05
	r RPFC	r PrG	−0.03 (0.22)	−0.09 (0.22)	−2.87	<0.05
	r RPFC	MCC	−0.03 (0.22)	−0.11 (0.20)	−3.31	<0.05
	l SMG	l PrG	0.08 (0.23)	0.01 (0.21)	−2.9	<0.05
	l SMG	r PrG	−0.13 (0.21)	0.04 (0.22)	−3.35	<0.05
SN-LN	r AI	r IFG	0.29 (0.25)	0.37 (0.25)	3.08	<0.05
	r RPFC	r IFG	0.04 (0.24)	0.11 (0.23)	3.16	<0.05
SN-CN	l RPFC	Anterior	−0.09 (0.18)	−0.04 (0.16)	2.77	<0.05
	r RPFC	Anterior	−0.09 (0.18)	−0.03 (0.18)	3.34	<0.05
SMN-FPN	MCC	l LPFC	−0.32 (0.19)	−0.24 (0.19)	3.18	<0.05
SMN-DAN	MCC	r IPS	0.3 (0.24)	0.21 (0.24)	−2.76	<0.05

Abbreviation: DMN, default mode network; FPN, frontoparietal network; SN, salience network; DAN, dorsal attention network; LN, language network; SMN, sensorimotor network; CN, cerebellar network; l, left; r, right; MPFC, medial prefrontal cortex; LP, lateral parietal; LPFC, lateral prefrontal cortex; PPC, posterior parietal cortex; AI, anterior insula; RPFC, rostral prefrontal cortex; IPS, intraparietal sulcus; PCC, posterior cingulate cortex; IFG, inferior frontal gyrus; FEF, frontal eyes field; dACC, dorsal anterior cingulate cortex; SMG, supramarginal gyrus, MCC, middle cingulate cortex; PrG, precentral gyrus. When the name of a network is used alone, it denotes the intra-network functional connectivity of that particular network; inter-network connectivity is denoted by joining the names of the respective networks with a hyphen.

## Data Availability

The data that support the findings of this study are available on request from the corresponding author. The data are not publicly available due to privacy or ethical restrictions.

## Supplementary Materials

The following supporting information can be downloaded at: https://www.mdpi.com/article/10.3390/jcm12093176/s1, Figure S1: The hot map of the partial correlation between the clinical variables and intra- and inter-networks; Table S1: The partial correlation between the clinical variables and intra- and inter-networks.

## Abbreviations

SCZ	Schizophrenia
HC	Healthy control
RSN	Resting-state network
FC	Functional connectivity
ROI	Region of interest
DMN	Default mode network
SN	Salience network
FPN	Frontoparietal network
CN	Cerebellar network
CEN	Central executive network
DAN	Dorsal attention network
SMN	Sensorimotor network
VN	Visual network
PANSS	Positive and Negative Syndrome Scale
MPFC	Medial prefrontal cortex
LP	Lateral parietal
PCC	Posterior cingulate cortex
PrG	Precentral gyrus
MCC	Middle cingulate cortex
dACC	Dorsal Anterior cingulate cortex
AI	Anterior insula
RPFC	Rostral prefrontal cortex
SMG	Supramarginal Gyrus
LPFC	Lateral prefrontal cortex
PPC	Posterior parietal cortex
IFG	Inferior frontal gyrus
PSTG	Posterior superior temporal gyrus
Anterior	Cerebellar anterior lobules
Posterior	Cerebellar posterior lobules
