# Supplementary material for: Widespread Intra- and Inter-Network Dysconnectivity among Large-Scale Resting State Networks in Schizophrenia
Source: J Clin Med. 2023 Apr 28;12(9):3176. doi: 10.3390/jcm12093176 (PMC10179370; doi:10.3390/jcm12093176)
Supplement: Supplementary file 1 [file jcm-12-03176-s001.zip › jcm-2213703-Supplementary.pdf]

Table S1. The partial correlation between the clinical variables and intra- and inter-networks.

	PANSS_total scores r(P)	PANSS_positive scores r(P)	PANSS_negative scores r(P)	PANSS_general scores r(P)	Duration of illness r(P)	CPZ r(P)
MPFC_l LP	-0.005(0.94)	0.053(0.465)	0.003(0.968)	-0.066(0.361)	-0.098(0.178)	0.006(0.937)
MPFC_r LP	-0.086(0.236)	0.009(0.898)	-0.007(0.927)	-0.167(0.02)	-0.086(0.236)	0.057(0.43)
Anterior lobe_Posterior lobe	-0.065(0.367)	-0.049(0.498)	-0.078(0.284)	-0.024(0.743)	-0.096(0.187)	-0.015(0.834)
MPFC_l LPFC	-0.082(0.255)	-0.041(0.57)	-0.047(0.515)	-0.095(0.189)	-0.039(0.591)	-0.051(0.482)
MPFC_r LPFC	-0.054(0.458)	-0.046(0.523)	-0.032(0.663)	-0.071(0.326)	-0.069(0.343)	-0.008(0.913)
MPFC_l PPC	-0.088(0.225)	-0.019(0.792)	-0.04(0.578)	-0.138(0.056)	-0.138(0.057)	0.074(0.306)
MPFC_r PPC	-0.098(0.176)	-0.081(0.267)	-0.082(0.261)	-0.087(0.233)	-0.049(0.496)	0.041(0.568)
MPFC_dACC	0.043(0.556)	0.003(0.969)	0.039(0.594)	0.032(0.662)	-0.045(0.534)	-0.077(0.291)
MPFC_l AI	0.02(0.78)	-0.077(0.29)	0.071(0.33)	0.05(0.49)	0.142(0.05)	-0.001(0.987)
MPFC_r AI	0.004(0.957)	-0.034(0.635)	0.062(0.395)	-0.019(0.797)	0.109(0.134)	0.032(0.659)
MPFC_l RPFC	0.019(0.792)	-0.017(0.81)	0.047(0.514)	-0.018(0.809)	0.055(0.451)	0.01(0.886)
MPFC_l IPS	0(1)	-0.031(0.672)	-0.022(0.76)	0.071(0.329)	-0.128(0.077)	-0.029(0.686)
MPFC_r IPS	-0.024(0.739)	-0.095(0.192)	-0.044(0.542)	0.068(0.348)	-0.11(0.13)	-0.044(0.547)
MPFC_l IFG	-0.032(0.663)	-0.038(0.605)	0.014(0.85)	-0.073(0.317)	0.073(0.318)	0.012(0.864)
PCC_l IFG	-0.061(0.4)	0.006(0.929)	-0.04(0.584)	-0.078(0.28)	-0.015(0.835)	0.001(0.992)
l LP_r IFG	0.084(0.248)	0.062(0.39)	0.04(0.585)	0.05(0.488)	0.006(0.929)	0.004(0.96)
r LP_r IFG	0.103(0.157)	0.044(0.54)	0.115(0.114)	0.02(0.785)	-0.052(0.474)	0.001(0.993)
l LP_MCC	0.047(0.519)	-0.035(0.627)	0.096(0.186)	0.035(0.633)	0.039(0.588)	0.073(0.317)
r AI_l IPS	-0.035(0.63)	0.069(0.339)	-0.045(0.532)	-0.074(0.307)	-0.147(0.042)	-0.026(0.722)
l AI_l PrG	0.027(0.705)	0.096(0.186)	0.035(0.632)	-0.051(0.484)	0.021(0.768)	-0.073(0.314)
l AI_r PrG	0.024(0.742)	0.098(0.178)	0.02(0.783)	-0.05(0.488)	0.072(0.324)	0.009(0.907)
l AI_MCC	0.015(0.842)	0.113(0.118)	-0.086(0.236)	0.024(0.737)	0.039(0.589)	-0.079(0.274)
r AI_l PrG	0.106(0.144)	0.068(0.351)	0.087(0.232)	0.046(0.524)	0(0.996)	0.05(0.49)
r AI_r PrG	0.051(0.485)	0.054(0.46)	0.061(0.4)	-0.014(0.844)	0.046(0.529)	0.121(0.094)
r AI_MCC	-0.024(0.737)	0.04(0.582)	-0.034(0.64)	-0.024(0.741)	0.022(0.758)	0.024(0.744)
l RPFC_l PrG	0.201(0.005)	0.071(0.328)	0.2(0.005)	0.15(0.038)	-0.003(0.963)	-0.027(0.713)
l RPFC_r PrG	0.152(0.036)	0.066(0.36)	0.144(0.047)	0.11(0.128)	0.04(0.581)	0.094(0.194)
l RPFC_MCC	0.124(0.086)	0.166(0.022)	0.033(0.648)	0.078(0.281)	0.064(0.379)	-0.071(0.328)
r RPFC_l PrG	0.151(0.036)	0.049(0.497)	0.162(0.025)	0.096(0.184)	0.062(0.395)	0.047(0.515)
r RPFC_r PrG	0.027(0.711)	0.016(0.83)	0.024(0.737)	0.011(0.876)	0.084(0.247)	0.13(0.073)
r RPFC_MCC	-0.003(0.971)	0.085(0.241)	-0.023(0.752)	-0.018(0.804)	0.077(0.286)	0.025(0.733)
l SMG_l PrG	0.063(0.387)	0.031(0.669)	0.063(0.382)	0.016(0.821)	-0.01(0.891)	0.162(0.025)
l SMG_r PrG	0.039(0.592)	-0.011(0.884)	0.088(0.225)	-0.015(0.831)	0.065(0.369)	0.188(0.009)
r AI_r IFG	-0.027(0.713)	0.05(0.489)	-0.002(0.982)	-0.059(0.417)	-0.01(0.891)	-0.048(0.508)
r RPFC_r IFG	-0.016(0.826)	0.015(0.839)	0.022(0.761)	-0.082(0.258)	0.031(0.669)	0.035(0.626)
l RPFC_Anterior lobe	-0.014(0.842)	-0.047(0.519)	0.001(0.987)	-0.011(0.885)	0.12(0.098)	0.027(0.711)
r RPFC_Anterior lobe	0.008(0.908)	-0.004(0.957)	-0.013(0.856)	-0.013(0.856)	0.057(0.433)	0.001(0.985)
MCC_l LPFC	-0.038(0.604)	0.098(0.175)	-0.135(0.062)	-0.034(0.637)	0.016(0.829)	0.09(0.214)
MCC_r IPS	0.061(0.403)	0.105(0.147)	-0.024(0.745)	0.046(0.528)	-0.087(0.23)	0.116(0.109)

Abbreviations: l, left; r, right; MPFC, medial prefrontal cortex; LP, lateral parietal; LPFC, lateral prefrontal

cortex; PPC, posterior parietal cortex; AI, anterior insula; RPFC, rostral prefrontal cortex; IPS, intraparietal sulcus; PCC, posterior cingulate cortex; IFG, inferior frontal gyrus; FEF, frontal eyes field; dACC, dorsal anterior cingulate cortex; SMG, supramarginal gyrus, MCC, middle cingulate cortex; PrG, precentral gyrus; PANSS, positive and negative symptom scale; PANSS_positive scores, positive symptoms scores; PANSS_negative scores, positive symptoms scores; PANSS_general scores, general psychopathology symptoms scores; CPZ, chlorpromazine equivalents.

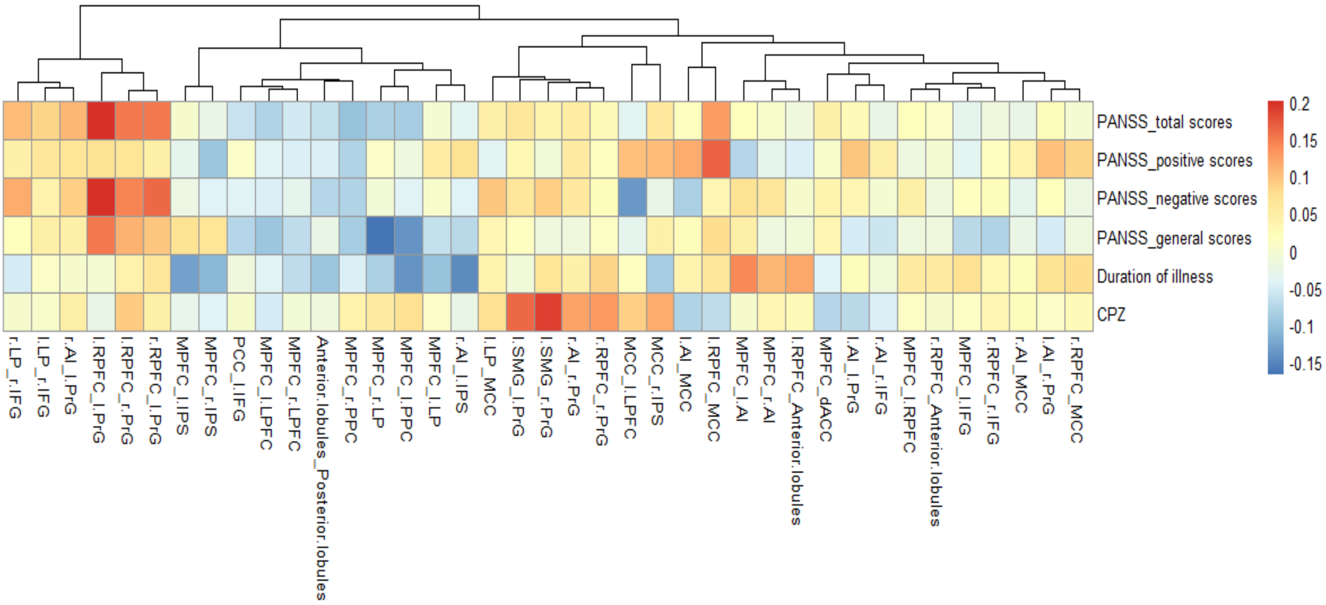

Figure S1. The hotmap of the partial correlation between the clinical variables and intra- and inter-networks. Abbreviations: l, left; r, right; MPFC, medial prefrontal cortex; LP, lateral parietal; LPFC, lateral prefrontal cortex; PPC, posterior parietal cortex; AI, anterior insula; RPFC, rostral prefrontal cortex; IPS, intraparietal sulcus; PCC, posterior cingulate cortex; IFG, inferior frontal gyrus; FEF, frontal eyes field; dACC, dorsal anterior cingulate cortex; SMG, supramarginal gyrus, MCC, middle cingulate cortex; PrG, precentral gyrus; PANSS, positive and negative symptom scale; PANSS_positive scores, positive symptoms scores; PANSS_negative scores, positive symptoms scores; PANSS_general scores, general psychopathology symptoms scores; CPZ, chlorpromazine equivalents.
